# Cleaning and Disinfection Protocols for Clear Orthodontic Aligners: A Systematic Review

**DOI:** 10.3390/healthcare10020340

**Published:** 2022-02-10

**Authors:** Carole Charavet, Zoé Gourdain, Léa Graveline, Laurence Lupi

**Affiliations:** 1 Université Côte d’Azur, Faculté de Chirurgie Dentaire, Département d’Orthodontie, 06800 Nice, France; 2 Centre Hospitalier Universitaire de Nice, Pôle Odontologie, 06000 Nice, France; zoe.gourdain@etu.univ-cotedazur.fr (Z.G.); lea.graveline@etu.univ-cotedazur.fr (L.G.); 3 Laboratoire MICORALIS UPR 7354, Université Côte d’Azur, 06800 Nice, France; 4 Université Côte d’Azur, Faculté de Chirurgie Dentaire, 06800 Nice, France; 5 Université Côte d’Azur, Faculté de Chirurgie Dentaire, Département de Santé Publique, 06800 Nice, France; Laurence.LUPI@univ-cotedazur.fr

**Keywords:** aligners, orthodontic aligners, biofilm, oral health care, cleaning protocols, disinfection protocols

## Abstract

(1) Background: Clear orthodontic aligners support the development of oral biofilms, which could lead to interferences with the oral microbiota already existing and the deterioration of oral health, with the development of dental caries, periodontal disease and even systemic infections. Therefore, preventive oral health care requires a cleaning and disinfection procedure for aligners. (2) Methods: A systematic review of the literature was conducted across four databases following the PRISMA guidelines up to May 2021, combining an electronic and a manual search. Prospective studies, including randomized controlled trials (RCTs), crossover studies (COSs) and controlled clinical trials (CCTs), published in the English language without time restrictions, evaluating the efficacy of cleaning and disinfection protocols for clear orthodontic aligners by comparing them with a placebo or a negative control, were included. The article selection, data extraction and risk of bias assessment were performed by two independent blinded review authors. In case of disagreement, a third author was solicited throughout the selection process. (3) Results: Among the 221 articles screened in the search process, 4 studies were included in the review, all designed as crossover studies (single arm without randomization with the same sequence of different cleaning and disinfection protocols for each participant). Different cleaning and disinfection methods were studied such as mechanical methods (brushing with toothpaste or vibration), chemical methods/pharmaceutical products (chlorhexidine antibacterial substance, anionic or cationic detergents or effervescent tablets) or combinations of both. (4) Conclusion: Although the determination of the most remarkable method of cleaning and disinfection was impossible because no direct comparison was conducted between all these methods, a multi-step protocol, including the combination of a mechanical and a chemical method, seems to be the most effective approach. Further research is needed to define the most preventive oral health care protocol. Registration: PROSPERO CRD 42021278498.

## 1. Introduction

Orthodontic procedures require the use of orthodontic appliances which can be fixed or removable, such as acrylic-based appliances or clear aligners. As aligners meet patients’ needs for discreet and comfortable orthodontic appliances, demand has increased over the past decade, and several studies have been carried out on clear aligners, including their clinical efficacy [1,2,3], but also their comfort in terms of social life and practicality [3,4,5,6].

Although clear aligner orthodontic treatment demonstrated encouraging results in terms of the plaque index and gingival health control compared to classic fixed orthodontic treatment [7,8,9], bacteria can form oral biofilms on the surface of the aligners. Indeed, Tektas et al. [10] demonstrated that the initial microbial adhesion and biofilm formation of aerobic and anaerobic oral species were similar between enamel, metal orthodontic brackets and aligner materials. Moreover, bacterial adhesion to aligners appears to be increased by the shape of the aligner, which contains grooves and ridges. Additionally, Low et al. [11] showed that the surface of the aligner itself tends to be corrugated, presenting scratch marks, microabrasions and peaks, even if it is new, and these irregularities are the starting point of bacterial adhesion and development. Additionally, Gracco et al. [12] demonstrated chemical and physical changes on aligners when they are worn: after 14 days, aligners showed microcracks and abraded and delaminated areas conducive to bacterial adhesion and growth, as well as localized calcified biofilm deposits and loss of transparency. Schuster et al. [13] also analyzed intra-oral aged aligners and found abrasion at the cusp tips, adsorption of integuments and localized calcification of the precipitated biofilm at stagnation sites. Finally, Low et al. [11] studied the ultrastructure and morphology of biofilms on aligners in “fast” and “slow” plaque formers. They showed that the initial biofilm (first 12 hours for slow plaque formers and first 6 hours for fast plaque formers) was composed of a majority of coccoid species. After this initial development, the bacterial biofilm develops into more diverse bacteria encased in a thick extracellular polymeric substance, with irregular calcifications. From another point of view, Zhao et al. [14] also studied the microbial changes during clear aligner treatment (CAT), comparing the oral bacterial communities of twenty-five patients before and 6 months after the beginning of the treatment. They identified the appearance of periodontal pathogens and cariogenic bacteria including *Aggregatibacter actinomycetemcomitans*, *Fusobacterium nucleatum*, *Treponema denticola*, *Porphyromonas gingivalis*, *Streptococcus mutans* and *Streptococcus sobrinus*, which were present all through the CAT. Finally, there is no self-cleaning process caused by the friction of the lips and the tongue on the teeth during CAT, which can lead to adverse effects.

Considering all the parameters mentioned above, the hygiene of the aligner appliances must be the sole object of the attention of the practitioner as well as patients. In this regard, it also seems relevant to investigate the different protocols of disinfecting/cleaning clear aligners to prevent biofilm formation. While a recent systematic review on this topic has been conducted for removable acrylic orthodontic appliances designed preferentially for children [15], to the best of our knowledge, this has never been carried out for orthodontic aligner appliances.

Therefore, the aim of this systematic review was to investigate, for the first time, the different protocols for cleaning and disinfecting clear orthodontic aligners.

## 2. Materials and Methods

### 2.1. Protocol

This systematic review (SR) was written following the guidelines of Preferred Reporting Items for Systematic Reviews and Meta-Analyses (PRISMA) as closely as possible [16]. This SR was registered in the International Prospective Register of Systematic Review (PROSPERO; CRD 42021278498).

### 2.2. Research Question and Eligibility Criteria

The review question was defined as “What are the cleaning and disinfection protocols for clear aligners?” In order to answer the review question, the “Population, Intervention, Comparison, Outcome and Study Design (PICOs)” format was employed, as follows: 

P: Population/Problem: Clear orthodontic aligners worn by orthodontic patients;

I: Intervention: Cleaning and disinfection protocols;

C: Controls: Placebo or negative control;

O: Outcomes: Bacterial biofilm; 

S: Study designs: All prospective studies, including randomized controlled trials (RCTs), crossover studies (COSs), and controlled clinical trials (CCTs), published in the English language without time restrictions, evaluating the efficacy of cleaning and disinfection protocols for clear orthodontic aligners by comparing them with a placebo or a negative control, were included. All retrospective studies, case reports/series, surveys, editorials, expert opinions, literature reviews and brief communications were excluded from the research. Additionally, animal experimental models and in vitro studies were also excluded.

### 2.3. Search Strategy

A search strategy was established from a combination of the National Library of Medicine’s medical subject headings (MeSH), entry terms and key words using Boolean operators to identify all peer-reviewed articles meeting the PICOs criteria in four electronic databases: PubMed, Scopus, Cochrane Central Register of Controlled Trials (CENTRAL) and Embase (Table 1). The search of the Scopus database was limited to articles regarding dentistry. In addition, the bibliography of each included paper was manually reviewed to find eligible studies, which were not initially revealed by the electronic searches.

### 2.4. Selection Methodology

Article selection was exported to a reference management software (Zotero version 5.0.96.2) and was conducted following 3 steps. First, duplicates were removed. Furthermore, the title and then abstract of the remaining records were evaluated by 2 independent authors, excluding all irrelevant references. Finally, the full text of all relevant articles was read to verify their eligibility. In case of disagreement, the decision to include or reject the article was made via discussion with a 3rd author throughout the selection process.

### 2.5. Data Extraction

The data extraction from all included papers was performed independently by two authors and presented parameters according to the previously defined PICOs, such as: study information (author, year and journal of publication), study design, population in terms of patients and in terms of aligners, inclusion and exclusion criteria, intervention’s description, type of comparison (control or placebo), the main variable studied, results and, finally, support. 

### 2.6. Risk of Bias Assessment

The evaluation of the risk of bias for eligible studies was performed independently by two authors, and in case of discrepancies, a third examiner was consulted. The risk of bias tool was chosen according to the type of study, as follows:

- Risk of bias in randomized controlled trials (RCTs) was assessed following the “Cochrane collaboration’s tool for assessing risk of bias in randomized trials” [17]. More specifically, for randomized crossover trials, the modified Rob-2 tool was used [18].
- For clinical controlled trials (CCTs), the risk of bias assessment was performed following the ROBINS-I (Risk of Bias in Non-randomized Studies-of Interventions) tool [19].

A “traffic light” plot of the domain-level judgements for each individual result was created with the robvis tool. A summary of the risk of bias frequency in the articles was also presented [20].

## 3. Results

### 3.1. Study Selection

**Figure 1:**
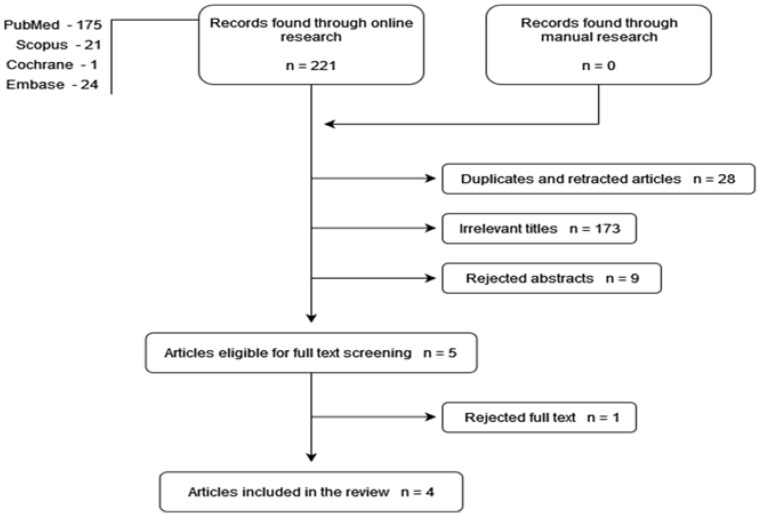
Flowchart diagram of the selection process according to the PRISMA (Preferred Reported Items for Systematic Reviews Analysis) method.

The search was conducted on 29 March 2021. A total of 221 articles (PubMed 175; Cochrane Library 1; Embase 24; Scopus 21) were initially identified after the electronic research. Removal of duplicates eliminated 28 articles. Selection by title and abstract resulted in the elimination of 173 and 9 articles, respectively, leaving 5 articles assessed for full-text eligibility. Reviewing the full text of these articles led to the exclusion of one article (did not correspond to the inclusion criteria). Thus, a total of four articles were included in the present systematic review [21,22,23,24]. A flowchart of the article selection procedure is described in Figure 1.

### 3.2. Study Characteristics

The four studies were published recently, between 2014 and 2020, from Israel [21] and Italy [22,23,24]. Of these four articles, all were designed as crossover studies (single arm without randomization with the same sequence of different cleaning and disinfection protocols for each participant). One study was supported by a grant from Align Technology [21].

These four studies shared a similar multi-step protocol: each participant had to successively perform the same sequence of different cleaning/disinfection protocols for their aligners. Samples were then collected and treated for analysis. 

Two types of aligners were examined featuring two types of manufacturing processes: printed [21,22,23] (Invisalign^®^, Align Technology, Santa Clara, CA, USA; material composition: multilayer aromatic thermoplastic polyurethane/copolyester), or molded [24] (aligners formed of thermoplastic polyurethane from resin models used as molds).

The disinfection and cleaning protocols analyzed were as follows:

- Brushing with toothpaste alone [23] or combined with chlorhexidine mouthwash immersion [21], or combined with Invisalign Cleaning System^®^ effervescent tablets (Align Technology) [22,23];
- Immersion in vibrating bath with Invisalign Cleaning-Crystal^®^ solution (Align Technology) [21];
- Immersion in anionic (TCS fresh^®^) or cationic (benzalkonium chloride, Caelo^®^) detergents [24] combined with a sonic (TCS Fresh^®^, 5800 Hz) or ultrasonic (iSonic F3900^®^, 42000Hz) bath [24].
- The different controls were as follows:
- Brushing with toothpaste [21];
- Rinsing under tap water [22,23];
- Immersion in water without vibration [24].
- The parameters studied were as follows:
- The quantification of the bacterial biofilm adhesion by spectrophotometric measurement [21];
- The quantity of plaque on the surface of the aligner by visual analysis in scanning electron microscopy (SEM) [22], a bioluminometer in SEM [23] or grey-scale measurement [24].

### 3.3. Risk of Bias Assessment

**Figure 2:**
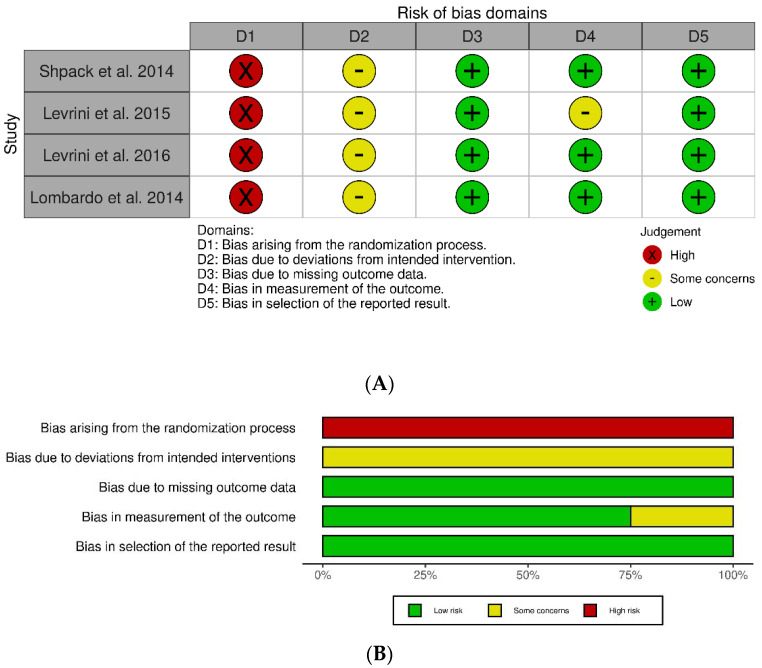
(**A**) Robvis traffic light plots of the risk of bias assessment of the studies according to the modified Rob-2 tool for crossover studies assessment tool. (**B**) Summary of the risk of bias per item from D1 to D5.

Since each study was designed as a crossover study, the modified Rob-2 tool was used (Figure 2A). This tool is designed for randomized crossover trials, and the first risk of bias parameter, which is “bias arising from randomization process”, was considered as high. The second represented risk of bias is the “deviation from intended intervention”, which was moderate in each study [21,22,23,24] because compliance bias was not controlled. Finally, the risk of bias in “measurement of the outcome” was considered as “some concerns” in one study [22] because the outcome of this study was based on visual observation (Figure 2B).

### 3.4. Data Extraction

As shown in Table 2 and Table 3, the synthesis was as follows: Concerning the mechanical methods such as brushing, according to Levrini et al. [22,23], brushing the aligner with toothpaste can efficiently remove the biofilm from the surface of the aligner, but it was not specified whether this result is statistically significant. Concerning the vibration mechanical method, in the study conducted by Lombardo et al. [24], sonic or ultrasonic vibration without a detergent was more efficient than an immersion in water without vibration (*p* < 0.05). By integrating a pharmaceutical product, the different studies demonstrated that brushing was more efficient if added to the chemical effect of a chlorhexidine mouthwash (*p* = 0.001) [21] or Invisalign Effervescent tablets (*p* = 0.0003) [22,23]. However, brushing with toothpaste and chlorhexidine is less performant than a vibrating bath with Cleaning Crystal solution (*p* = 0.001) [23]. Additionally, Shpack et al. [24] demonstrated that, among all the overall combinations possible (sonic bath/ultrasonic bath/no bath combined with anionic detergent/cationic detergent/no detergent), ultrasonic vibration associated with a cationic detergent was significantly the most effective protocol (*p* < 0.05). Interestingly, Shpack et al. [24] also showed that the cationic detergent or the anionic detergent could efficiently remove the biofilm even without sonic or ultrasonic vibration (*p* < 0.05).

## 4. Discussion

Although removable orthodontic clear aligner appliances can be removed for eating, drinking and performing oral hygiene routines, the adhesion and growth of bacterial biofilms on these appliances are possible. The consequences are multiple such as deterioration of the esthetic appearance and a disagreeable odor, which could also lead to a decrease in patient compliance. Considering the capacity of oral biofilms to expand and spread in the mouth, the presence of a bacterial biofilm on the aligner’s surface could lead to interferences with the oral microbiota already existing and the development of bacteria-related diseases such as dental caries, periodontal disease [25] and even systemic infections [26]. Therefore, this problem should be not ignored; thus, this systematic review was designed, for the first time, to investigate cleaning and disinfection protocols for clear orthodontic aligners which were studied by comparing them with a placebo or a negative control.

First of all, Levrini et al. demonstrated that brushing with toothpaste alone is more efficient than rinsing under tap water, although better results are obtained when brushing with toothpaste is combined with an effervescent tablet [22,23]. Therefore, the addition of effervescent tablets to brushing with toothpaste increased cleaning efficacy. The tablets used in these studies contained sodium carbonate and sulfate, but the antibacterial effect of these products has not been demonstrated. This raises the question of whether the antibacterial effect of the tablets was due to the chemical itself, the mechanical action of the bubbles or a synergy of both.

However, whether an effervescent tablet was added or not, the biofilm was not completely eradicated [22,23]. Therefore, the brushing technique itself seems to be an important factor to control biofilm formation, and this concern was also highlighted in the discussion by the same authors [22]. Indeed, according to the authors, patients should be advised to pay attention to cleaning not only the external surface but also the internal surface, which seems to receive less attention from patients [22]. In addition, the internal surface appears to be more difficult to brush perfectly, notably due to the many hollows that are difficult to access while being necessary for the attachments, which makes cleaning difficult. Additionally, Shpack et al. [21] demonstrated that the posterior palatal and the anterior incisal edge regions had significantly more biofilm adherence than the anterior region of the aligner. Interestingly, as with teeth, more plaque is found in the posterior area than in the anterior area. On the other hand, the buccal surface of the teeth is the surface most subject to plaque accumulation, whereas it is the palatal surface on aligners. This could be explained by the absence of the tongue and mucosa self-cleansing which occurs on the teeth in the absence of aligners. Furthermore, according to the discussion of Shpack et al. [21], the incisive edge collects and retains all food debris. Finally, aligner wear leads to chemical and physical changes on the aligners, which also reduce the usefulness of careful and precise brushing [12]. Therefore, it seems very important to inform and educate the patient about the importance of proper mechanical cleaning of their aligners to avoid initial microbial adhesion and biofilm formation. In this perspective, providing information to our patients about the anatomical characteristics of aligners, and showing them the presence of cavities, conducive to plaque retention, seem relevant. Finally, some motivational methods such as those already used for fixed orthodontic patients have already shown good results [27] and seem to be adaptable to clear aligner treatment (CAT).

Concerning the use of vibration, according to Lombardo et al. [24], a sonic or ultrasonic bath was significatively more efficient in removing bacterial biofilms from aligners than an immersion in water without vibration. Therefore, vibration alone seems to be a sufficient method to remove biofilms on aligners. Lombardo et al. [24] found that there was significantly less biofilm at the surface of the aligners after their immersion in a cationic or anionic detergent than in water only. Therefore, the disinfectant effect of these chemicals seems acceptable for the cleaning of the aligners. However, the addition of the cationic detergent with ultrasonic vibration was significantly superior to any other combination. Therefore, the combination of these two parameters produces a synergetic effect, which increases the efficiency of the cleaning strategy. However, the SEM pictures obtained showed that the surface of the thermoplastic polyurethane was damaged by the ultrasonic vibrations, showing clear signs of water absorption and ultrasonic cavitation [24]. This damage could have severe consequences, i.e., increasing the potential bacterial adhesion because of the irregularities of the surface. As such, Ihssen et al. demonstrated that water absorption can reduce the material’s Young’s modulus and may therefore promote a decrease in the resulting orthodontic forces in PolyEthylene Terephthalate Glycol (PETG) aligners [28].

Furthermore, in the study conducted by Shpack et al. [21], a vibrating bath with Cleaning Crystal solution (Align Technology ^®^) was significantly more effective than brushing with toothpaste combined with chlorhexidine. However, in the in vitro section of this article, which was not included in this SR according to the PICO format, they found that the Cleaning Crystal solution alone (Align Technology ^®^) demonstrated no bacterial inhibition zone. Therefore, it appears again that the vibration bath plays a prominent role in removing biofilm and could possibly be more effective than brushing with toothpaste combined with chlorhexidine. An additional point is also worth discussing: the Cleaning Crystal solution (Align Technology ^®^) itself is composed of microcrystals, and thus the solution might have a mechanical action itself, with the impact of these crystals on the surface of the aligner induced by the vibration. However, according to the manufacturer’s recommendation’s, the user has to add one packet of Cleaning Crystals to water and “Gently shake/agitate for 20 s to dissolve and distribute crystals”. Then, the aligner should be immersed in water for 20 min without vibration, except for a 20 s agitation of the solution after the waiting time. This procedure seems to contradict the idea of the solution having a mechanical effect, and if this solution does not have any antibacterial or mechanical effect, it would be interesting to investigate its role alone. As the composition of the Cleaning Crystals used in the vibrating bath is composed of Sodium Sulfate (60%), Sodium Carbonate (30%), Sodium Tripolyphosphate (7.5%), Sodium Dichloroisocyanurate (2%) and Sodium lauryl sulfate (0.15%), which act as a pH neutralizer (e.g., Sodium Sulfate), disinfectant (e.g., Sodium Dichloroisocyanurate) and detergent (e.g., Sodium lauryl sulfate) [29], some additional investigations may be necessary.

Regarding the significantly inferior effect of the group using chlorhexidine (brushing with toothpaste combined with chlorhexidine versus a vibrating bath with the Cleaning Crystal solution) (Align Technology ^®^), some points could also be discussed. In fact, while chlorhexidine is recognized as the gold standard compared to other antiplaque and anti-gingivitis agents due, in part, to the persistence of its effect [30], Shpack et al. [21] showed that the aligner material does not absorb chlorhexidine in an in vitro study, and thus its persistent effect cannot occur. This result is consistent with the study conducted by Schaefer et al. [4]: in a crossover study, they evaluated the impact of a low-dose chlorhexidine mouthwash on periodontal health, halitosis and quality of life during clear aligner treatment. They concluded that there was no value in generally recommending the addition of this mouthwash to patients’ oral hygiene procedures. In addition, this disinfectant is known to cause staining on several surfaces, including the teeth [31]. However, aligners are susceptible to staining from certain beverages, including coffee, black tea and red wine [32,33]. Therefore, the staining effect of chlorhexidine could occur on aligners, which would be a problem for patients seeking invisible orthodontic treatment.

It is interesting to note that this topic is very stimulating in terms of research because innovative techniques can also be highlighted. The study conducted by Meto et al. [34], which was removed from this systematic review due to its case report design, tested the efficacy of Cupral ^®^, a copper-calcium hydroxide solution, as a disinfectant on a biofilm-contaminated aligner. This is an innovative method; its effectiveness has been demonstrated in periodontal or endodontic infections [35,36], but never in orthodontics, and its mechanisms of action remain unknown. In this study, Cupral ^®^ showed encouraging results in terms of elimination of aerobic and anaerobic bacteria. In addition, the studies conducted by Zhang et al. [37] and Xie et al. [38], which were removed from the review because they were designed as in vitro studies, incorporated a gold nanoparticle coating on the surface of the aligner, which controls the development of the biofilm by preventing the initial adhesion of bacteria. The in vitro results were very encouraging, since they showed an inhibition of *Porphyromonas gingivalis* [37] and *Streptococcus mutans*, [38], a periodontopathogenic bacterium and a cariogenic bacterium, respectively. Moreover, the incorporation of an antibacterial substance into the biomaterial to inhibit bacterial growth has already been used in acrylic-based orthodontic appliances, with good results [39].

Finally, some points also deserve to be addressed. First of all, in addition to the cleaning and disinfecting action, the protocol must preserve all the mechanical and chemical properties of the aligner and not undergo any degradation. In addition, its color must remain stable. Finally, the malodor parameter, which patients report after wearing the aligners, must also be considered. Indeed, the cleaning and disinfection product must also be able to neutralize any unpleasant odor. Moreover, if a multi-step protocol is chosen, the patient’s compliance to such a protocol must be evaluated because it will generate an additional cost and take a relatively long time. Indeed, it seems difficult to implement a multi-step protocol at each meal. These parameters are not considered in the included studies. Additionally, two types of aligners were examined, with two types of manufacturing processes, which could be considered a confounding factor. Finally, few studies were included in this systematic review (note that only articles published in English were included), and all were designed as crossover studies (single arm without randomization with the same sequence of different cleaning and disinfection protocols for each participant). However, surprisingly, no period of “wash-out” between the different protocols was performed in any study, including those using a chlorhexidine mouthwash. In the study of Solis et al. [40], which investigated the level of staining and clinical efficacy of a chlorhexidine mouthwash with an antidiscoloration system versus traditional chlorhexidine, a 15-day wash-out period was applied between the use of these two products. All in all, no study designed as a randomized controlled trial was found, leading to further interesting and stimulating investigations.

## 5. Conclusions

Based on this first systematic review, different cleaning and disinfection protocols seem to be able to control the adhesion and development of biofilms on the surface of aligners. The combination of a mechanical and a chemical method seems to be the most effective approach. Specifically regarding the mechanical method of brushing the aligner with toothpaste, it is very important to perform it in a precise and careful way, including all surfaces and edges of the aligner. Particular attention should be paid to the posterior palatal region and the incisal edge, which present the greatest biofilm formation. This information should be communicated to the patient.

The data available in the studies included in this systematic review do not provide enough evidence to determine the most effective cleaning and disinfection method. Additional investigations, designed as randomized controlled trials, would be very useful in order to more deeply explore this relevant topic.

## Figures and Tables

**Table 1:** Search strategy.

Database	Keywords
Pubmed	(((Orthodont* OR Clear) aligner*) OR Invisalign OR (removable thermoplastic (appliance OR aligner))) AND (((removal OR reducing OR controlling) AND (biofilm OR bacteria OR “dental plaque”)) OR disinfection OR decontamination OR “Disinfection/methods”[MeSH] OR “Biofilms/drug effects”[MeSH] OR “Equipment Contamination/prevention and control”[Mesh] OR “Anti-Infective Agents, Local”[Mesh] OR “Oral Hygiene”[MeSH] OR antimicrobial OR antibacterial OR Clean*)
Scopus	(((Orthodontic OR Clear) AND aligner OR Invisalign OR (removable AND thermoplastic AND (appliance OR aligner))) AND (((removal OR reducing OR controlling) AND (biofilm OR bacteria OR dental plaque)) OR decontamination OR disinfection OR antimicrobial OR antibacterial OR clean*) AND (LIMIT-TO (SUBJAREA,”DENT”)) AND NOT (fixed OR bracket)
Embase, Cochrane	((Orthodont* OR Clear) aligner* OR Invisalign OR (removable thermoplastic (appliance OR aligner))) AND (“biofilm removal” OR “biofilm adhesion” OR “decontamination” OR “disinfection” OR “dental plaque” OR “antimicrobial” OR “antibacterial” OR “clean*”)

**Table 2:** Data extracted from the studies using the PICO approach. * Crossover studies (single arm without randomization with the same sequence of different cleaning and disinfection protocols for each participant). NSAID: non-steroidal anti-inflammatory drugs.

Author Year	Journal	Study Design	Population	Inclusion Criteria	Exclusion Criteria	Intervention	Comparison	Support
**Shpack et al. ** **2014**	*Angle* * Orthodontist*	Crossover Study *	11 participants; 132 printed aligners (Invisalign^®^, Align Technology)	-	-	- Brushing with toothpaste + chlorhexidine mouthwash - Vibration with Cleaning Crystal solution	Brushing with toothpaste	Grant from Align Technology
**Levrini et al.** **2015**	*Clinical, Cosmetic and Investigational Dentistry*	Crossover Study *	12 participants; 72 printed aligners (Invisalign^®^, Align Technology)	Good oral and systemic health. No caries or periodontal diseases. Patients who are candidates for orthodontic aligners.	-	- Effervescent tablets (Invisalign Cleaning System) + brushing with toothpaste - Brushing with toothpaste only	Rinsing under tap water	-
**Levrini et al.** **2016**	*International Journal of Dentistry*	Crossover Study *	20 participants; 120 printed aligners (Invisalign^®^, Align Technology)	Class I skeletal relationship normodivergent. Frankfort mandibular plane angle. Age > 18 years. No active periodontal disease.	Smoking habits, presence of fixed bridges/crowns or partial dentures, periodontal nonsurgical treatments < 1 year, medication (antibiotics, steroids or NSAID < 6 month)	- Brushing with toothpaste - Effervescent tablets (Invisalign Cleaning System) + brushing with toothpaste	Rinsing under tap water	-
**Lombardo et al.** **2016**	*Progress in* * Orthodontics*	Crossover Study *	5 participants; 90 molded aligners (formed of thermoplastic polyurethane from resin models used as molds)	-	-	- Immersion in a cationic or anionic detergent and sonic or ultrasonic bath and all the possible combinations.	Immersion in water without vibration	-

**Table 3:** Protocols, outcomes and results of included studies. SEM: scanning electron microscopy.

Author Year	Protocol	Variable	Results
**Shpack et al. ** **2014**	Each participant had to perform 3 different cleaning/disinfection protocols for their aligners successively: - Brushing with 1400 ppm fluoride toothpaste (control group); - Brushing with toothpaste + 15 min immersion in a chlorhexidine mouthwash (CHX group); - 15 min vibrating bath with Cleaning Crystal solution (VBC, Align Technology) (VBC group). Each protocol lasted for 2 weeks.	Bacterial biofilm adherence (photodensitometer measurement, arbitrary unit)	CHX and VBC groups showed a significant decrease in bacterial adhesion of 16% and 50%, respectively (*p* < 0,001), leading the VBC protocol to be 3 times more efficient than the CHX protocol. Under regular brushing with toothpaste, in the aligner, the posterior palatal region (premolar/molar) compared to the anterior region (central and lateral incisors) and the incisal edge compared to the incisal and middle region had the greatest plaque accumulation.
**Levrini et al.** ** 2015**	Each participant had to perform 3 different cleaning/disinfection protocols for their aligners successively: - 15 s rinsing under tap water (control group); - 30 min immersion in water with an effervescent tablet (Invisalign cleaning System, Align Technology, San jose, CA, USA) + 30 s brushing with a soft brush and a non-abrasive toothpaste (T&B group); - 30 s brushing with a soft brush and a non-abrasive toothpaste (brushing group). Each protocol lasted for 2 weeks.	Plaque quantity (SEM, visual evaluation)	Regarding the external surface of the trays, although the brushing group showed a better cleaned surface compared to the control group, the cleanest surface was found in the T&B group. Regarding the interior surface, no appreciable differences were observed between the three groups. The contamination was mainly organic (occasional inorganic and crystalline tartar), and a unique species of spheroidal microorganisms was found and growing in immense colonies, particularly manifest on the interior surface.
**Levrini et al.** ** 2016**	Each participant had to perform 3 different cleaning/disinfection protocols for their aligners successively: - 15 s rinsing under tap water (control group); - 30 s brushing with a soft brush and a non-abrasive toothpaste (brushing group); - 20 min immersion in water with effervescent tablets containing sodium carbonate and sulfate (Invisalign Cleaning System, Align Technology, San Jose, CA, USA) + 30 s brushing with a soft brush and a non-abrasive toothpaste (T&B group).Each protocol lasted for 2 weeks.	Bacteria concentration (bioluminometer analysis, relative light unit)	The mean value of the bacterial concentration was higher in the control group (585 RLU) compared to the brushing group (188 RLU) and the T&B group (71 RLU). These results are similar in terms of median value and 95% confidence interval, which were 518 RLU (interval 248–781) in the water group, 145 RLU (interval 103–205) in the brushing group and 64 RLU (interval 39–85) in the T&B group. The difference in bacterial concentration was only significant in the T&B group compared to the brushing group (*p* = 0.0003).
**Lombardo et al.** **2017**	Each participant had to perform 9 different cleaning/disinfection protocols of their aligners successively: - Rinsing under water (control); - Under the conditions to be immersed in water, all the combinations possible with these parameters: sonic bath (TCS Fresh, 5800 Hz), ultrasonic bath (iSonic F3900, 42000 Hz), anionic detergent (TCS fresh), cationic detergent (benzalkonium chloride, Caelo). Each protocol lasted for 2 weeks.	Bacterial biofilm observation (SEM with grey-scale measurements)	All cleaning strategy variables, except rinsing under tap water without vibration, had a significant effect on the SEM value (i.e., the “cleanliness” of the aligner). Interestingly, two methods were significantly different from the others: - Rinsing with water, which was the least efficient; - Immersion in water and cationic detergent in an ultrasonic bath, which was the most efficient (*p* < 0.05).

## Data Availability

The data underlying this article are available in the article.
